# A reverse pattern in work motivation among Vietnamese health care workers during the prolonged COVID-19 outbreak of 2021: Determinants and implications

**DOI:** 10.7189/jogh.13.06022

**Published:** 2023-05-26

**Authors:** Linh Phuong Doan, Bach Xuan Tran, Pascal Auquier, Laurent Boyer, Guillaume Fond, Toan Van Ngo, Minh Ngoc Vu Le, Giang Thu Vu, Thao Phuong Hoang, Phuong Thu Ho, Tu Huu Nguyen, Linh Khanh Le, Carl A Latkin, Roger C M Ho, Cyrus S H Ho

**Affiliations:** 1 Institute for Preventive Medicine and Public Health, Hanoi Medical University, Hanoi, Vietnam; 2 EA 3279, CEReSS, Research Centre on Health Services and Quality of Life, Aix Marseille University, Marseille, France; 3 Institute of Health Economics and Technology, Hanoi, Vietnam; 4 National Centre for Youth Substance Use Research, The University of Queensland, Brisbane, Australia; 5 Action Aid Vietnam, Hanoi, Vietnam; 6 Vietnam Young Physician Association, Hanoi, Vietnam; 7 Troy University, Troy, Alabama, USA; 8 Bloomberg School of Public Health, Johns Hopkins University, Baltimore, Maryland, USA; 9 Department of Psychological Medicine, Yong Loo Lin School of Medicine, National University of Singapore, Singapore, Singapore; 10 Institute for Health Innovation and Technology (iHealthtech), National University of Singapore, Singapore, Singapore

## Abstract

**Background:**

The workload burden of the COVID-19 pandemic on health systems requires not only financial support but also long-term and contextualized policies. We assessed the work motivation and its determinants among health workers at Vietnamese hospitals and facilities during the prolonged COVID-19 outbreaks in 2021.

**Methods:**

A cross-sectional study was conducted among 2814 health care professionals across all three regions of Vietnam from October to November 2021. An online questionnaire, including the Work Motivation Scale, distributed by the snowball method to a subgroup of 939 respondents, investigated changes in working characteristics due to COVID-19, work motivation, and occupational intention.

**Results:**

Only 37.2% of respondents committed to their current job and about 40% reported a decrease in their job satisfaction. The Work Motivation Scale scored the lowest in “financial motivation” and the highest in “perception of work value”. Participants who were in the north region, of younger age, unmarried, and who had a low level of adaptability to external work pressure, shorter experience, and less job satisfaction tended to be less motivated and committed to their current job.

**Conclusions:**

Intrinsic motivation has increased in importance during the pandemic. Therefore, policymakers should develop interventions that raise intrinsic, psychological motivation instead of only focusing on salary raises. Issues about intrinsic motivations of health care workers such as low adaptability to stress and professionalism in routine work should be prioritized during the pandemic preparedness and control.

Since the first outbreak in 2019, the coronavirus disease (COVID-19) pandemic has put the world in an unprecedented health crisis [1]. As of 2022, COVID-19 has caused 6 307 857 deaths and infected 530 350 724 people [2]. In Vietnam, a total of 10 714 008 cases were recorded in three years, equalling approximately 11% of the Vietnamese population. As it has with all other aspects of life, COVID-19 has completely disrupted the dynamic of health care professions. As the frontline in our fight against COVID-19, health care workers were inarguably the most affected group under the pandemic. Impacts on health care workers include amplified risks of infection, threats to health status, lack of personal protective equipment, and shortage of financial resources [3,4]. Beyond the individual level, COVID-19 health care workers also have to face social stigma, separation from family, and decreasing work value [5-7]. Previous studies conducted during major infectious diseases such as SARS and MERS-CoV found devastating impacts on the work motivation of health care workers [8,9]. Without proper schedule management, health care workers experienced serious burnout, fatigue, and loss of motivation which ultimately resulted in high turnover rates [10,11]. The extent of COVID-19 damage is not any less in severity, if not even more extreme compared to past pandemics. To date, COVID-19 is accountable for more deaths than SARS and MERS combined and is predicted to continue for at least another eight months, making it one of the longest pandemics in history [12,13]. Therefore, resolving internal problems to ensure the quality of the workforce is as vital and urgent as any effort of public safety measures during such a health emergency.

Work motivation is defined as the internal or external force affecting one’s work-related behaviour, shaping its form, intensity, and duration, and has significant correlations with one’s work performance [2]. Although work motivation is one of the prime indicators of workforce status [14], it has not received adequate attention in the health sector worldwide. Most studies of work motivation in health care before 2020 have focused on nurses and chronic caregivers who accounted for the majority of the workforce and were most likely to experience a shortage of motivation due to repetitive and rigorous working schedules [15]. Beginning in 2020, frontline COVID-19 health workers have replaced nurses to become the most vulnerable group within the workforce, subject to severe burdens in workload, socioeconomic state, and mental health. During this time, financial incentives were often considered the most effective motivator and have been adopted by many countries to encourage their overwhelmed workforce [16,17]. However, monetary incentives alone cannot resolve all existing problems of demotivation and overturn among health care workers – work motivation is a complex and interdisciplinary process, crossing areas such as economics, psychology, organization, and resource management.

Various frameworks have strived to identify determinants of work motivation and their correlations. A conceptual framework developed by Legesse Chala [18] divided motivation into four groups: 1) sociodemographic, 2) internal – responsibility, achievement, recognition, 3) external – interpersonal relation, justice and fairness, workplace characteristics, and 4) resource availability – medical supply, personal protective equipment (PPE) and drug provision. Another framework proposed by Banteyerga [19] divided motivations into two categories: 1) “will do” – societal values, personal values, personality characteristics, organization structure, financial rewards, and recognition, and 2) “can do” – self-perception of work, work orientation and self-regulatory skills. Among tens of conceptual frameworks proposed, basic determinants of work motivation included in this study such as demographics, job perception, or financial motives may have been introduced elsewhere. However, in all models proposed, no factor was able to account for the sudden surge of workload and the immediate, multilevel changes brought about by a large-scale emergency like a pandemic. Such unique characteristics of COVID-19 make past frameworks not entirely applicable to our current context. Our study, therefore, is the first to incorporate COVID-19-specific variables, namely the duration of participation in COVID-19 tasks, ability to deal with work pressures, or professionalism of routine tasks. While other factors such as community stigma, ability to complete assigned tasks, or complexity in coordination between colleagues are not necessarily novel, they were often considered minor by past assessment models. The scope of these factors has now been severely amplified by COVID-19 and therefore will be among the primary subjects for evaluation in this study.

In Vietnam, there is a wealth of literature attempting to investigate the general impacts of the pandemic on health care workers. However, none has been able to provide an in-depth analysis of their employment characteristics and the workforce’s response to the increasing challenges and demands. This gap in the literature has had immediate effects on the Vietnamese health workforce, as indicated by a dramatic surge in turnover rate during 2021. In the last three months of 2021, 400 health care workers in Ho Chi Minh City and hundreds in other regions of Vietnam have quit their job [20,21]. Understanding the burden of COVID-19 on health care work motivation is, therefore, ever more critical for adjustment in health policy and the development of a sustainable workforce. The purpose of this paper is to investigate the prevailing trends in work motivation among health care workers during the COVID-19 pandemic, as well as scrutinize such factors that determine it, and propose potential policy implications based on the findings.

## METHODS

### Study design and sampling method

A national online survey was carried out from October 2021 to November 2021 during the peak of the fourth COVID-19 wave to assess the impacts of COVID-19 on various aspects of life among Vietnamese people. Link to the questionnaire created by the online platform – Survey monkey to target subjects across different regions of Vietnam (surveymonkey.com). This survey was conducted in three groups: 1) health professionals; 2) medical students; 3) general population. For health professionals group, we focused on three topics of interest, including a) work motivation and retention; b) quality of life; c) burn out and mental well-being. After answering general social demographic questions, each respondent was randomly assigned to one of these three topics, generating three separate data sets.

In this target group, we included participants who 1) aged 20 or older; 2) agreed to participate in this study by providing online informed consent; 3) worked at medical facilities in Vietnam in the last six months; 4) could access the survey link and complete the questionnaire. The snowball sampling method was applied to collect and speed up the data collection process. At the end of the data collection duration, a total of 2814 health care professionals worked at medical facilities from 61 / 63 provinces in Vietnam. The substudy of work motivation among Vietnamese health care workers included 939 complete records.

### Procedure and instruments

To develop research instruments, we based on a standard procedure. First, a systematic review was conducted to explore gaps and important facets that have emerged from previous studies. Second, we developed an instrument that covers all topics of interest. Then, we invited experts in COVID-19 fields including public health practitioners, infectious diseases experts, health services providers, and policymakers who are representative of target groups to jointly deliberate throughout translating, rephrasing, piloting, and shortening the questionnaire. Finally, four main sections for this study were included in the questionnaire: 1) socio-demographic characteristics; 2) work characteristics; 3) the impact of COVID-19 on work; and 4) work motivation and commitment.

Before collecting the data, this questionnaire for the medical staff group was piloted by 20 staff members of the Vietnam Young Physicians Association (including medical doctors, nurses, technicians, and administrative staff) to test the language, logical order, text, and any technical issue one more time. After the questionnaire had been edited and launched, the above test data will be removed. In the next stage, a questionnaire link was sent to a small group of 30 medical staff. In particular, we selected 10 health professionals from each region of Vietnam (Northern, Central, and Southern) who worked in different types of health facilities (general hospitals, specialized hospitals, central clinics, and commune health stations) as the core facilitator group. Participants took 30 minutes to complete the questionnaire. Respondents were then invited their acquaintances and colleagues to participate in the survey. Participants were informed of the benefits and risks of participating in this study. Data are monitored and tracked through Survey Monkey's system. The data of the study was confidential and was used for research purposes only. The data used for this paper was extracted from the mentioned online survey. The study protocol was granted by the scientific committee of the YRI (Code: ĐT.KXĐTN22-11).

### Primary outcomes

#### Work motivation scale for health workers

Fifteen questions were adapted from the work motivation scale for health workers [22]. This scale was constructed into four domains corresponding to four aspects of work motivation including intrinsic motivation (three items), perception of work value (four items), societal motivation (four items), and financial motivation (four items). For each item, an 11-point Likert scale (from 0 = totally unimportant to 10 = totally important) was applied. Then, the total score of each domain was calculated by summing and converting to a scale from 0-40 points (for the intrinsic motivation domain). The Cronbach’s alpha of four domains and total scale was 0.91, 0.86, 0.85, and 0.87, The detail of the work motivation scale was described as follows:

Domain 1: intrinsic motivation: 1) because I enjoy doing what I do at work every day; 2) because I enjoy my work tasks; 3) because the work that I do is very interesting.

Domain 2: perception of work value: 4) because being a health worker is a fundamental part of who I am; 5) because my work is extremely important for my patients; 6) because I want to make a difference in people’s life; 7) In order to feel good about myself.

Domain 3: societal motivation: 8) because my reputation depends on my work; 9) because of the appreciation I receive from my patients and the community; 10) so I do not let my team down; 11) because my supervisor recognizes and appreciates me.

Domain 4: financial motivation: 12) because of the benefits that come with my job; 13) in order to be able to provide for my family; 14) because of the financial security my job provides me with; 15) in order to earn money.

A short questionnaire was designed to assess the participants’ occupational intention including five items: 1) continue to stick with the job at the current unit and be determined to complete the task with the highest ability; 2) consider transferring to another unit in the same organization; 3) consider transferring to another agency; 4) consider switching to another major in the health sector; 5) consider moving into a field other than the health sector.

#### Commitment to the current job

The questions asked participants to rate their certainty with each statement. Commitment to the current job is defined based on question 1 above (continue to stick with the job at the current unit and be determined to complete the task with the highest ability). For this question, a Likert-5 scale was applied from 1-certainly not to 5-definitely yes.

#### Changes in job satisfaction and job motivation

Furthermore, we developed to questions to assess the impact of COVID-19 on job satisfaction and job motivation of the participants, with answer options including dramatically decrease, slightly decrease, same, slightly increase, significantly increase.

### Covariates

Socio-demographic characteristics included following information: age group (21-30 years old / 31-40 years old / 40 or older), gender (male / female), level of education (intermediate or college / graduated / post-graduated), marital status (married / others), living area (city / town / rural or mountains), housing status (private house with parents / private house without parents / rented house or others), household economic conditions (under five million Vietnamese dong (VND) / 5-10 million VND / 10 million VND or above).

Work characteristics: information about types of facilities (general hospital, specialized / private hospital, centers for disease control and prevention (CDC) / medical centre / public health station, University hospital, other), level of facilities (central line, province / city line, district line, others), specialty (medical doctor, nurse / midwife, technician / administrative staff, others), contract status (civil servant, indefinite-term labour contract, others), working experience (under five years, five years to under 10 years, above 10 years), working seniority (under five years, five years to under 10 years, above 10 years), number of duty per week (none, one day, two days, three days and above), working time (under eight hours, eight hours, 9-10 hours, 11-12 hours, above 12 hours), part-time job (none, one, two and more), and duration of participation in the fight against COVID-19 (none, one-three months, 3-12 months, above 12 months).

Impact of COVID-19 on work: to assess the impact of COVID-19 on aspects of work among health professionals, 10 questions were developed to assess the changes in their work. All items were designed on a 5-point Likert scale (1 = completely unchanged; 2 = changed little; 3 = changed relatively much; 4 = changed a lot; 5 = changed extremely beyond processing capacity). The detail of these questions was presented as follows: 1) daily work intensity; 2) level of work-related stress and fatigue; 3) health risks caused by work; 4) the community's stigma with the work I'm doing; 5) your ability to endure and cope with external work pressures; 6) process and professionalism of routine work; 7) complexity in coordination between colleagues, and between departments; 8) new knowledge and skills for work; 9) ability to complete assigned tasks; 10) ability to ensure safe means of work.

### Data analysis

Data analyses were performed using STATA version 15 (Stata Corp. LP, College Station, United States of America). The Listwise Deletion method was applied to clean all missing data before analysing process. For each quantitative variable, Skewness and kurtosis tests (sktest) were used to assess the distribution. In particular, sktest presents a test for normality based on skewness and another based on kurtosis and then combines the two tests into an overall test statistic. Descriptive analysis was applied for all variables. Quantitative variables were presented as mean and standard deviation (SD) for normal distribution variables and median and interquartile range (IQR) were presented for non-normal distribution variables, while categorical variables were presented as frequencies and percentages.

Reliability: the internal consistency reliability was checked by calculating Cronbach's alpha. The Cronbach's alpha value of 0.7 or above was considered acceptable. In terms of work motivation, Cronbach’s alpha of the four domains and total scale was 0.91, 0.86, 0.85, and 0.87.

Principal components analysis: The Exploratory Factor Analysis (EFA) using principal component analysis (PCA) was applied to identify the optimal structural model of the work motivation instrument according to the observed data. The number of factors was determined based on the eigenvalues and the proportion of variance explained [23]. Items with a loading value ≥ of 0.4 were included in the relevant components [23]. After applying EFA, the optimal structural model of work motivation has four domains including intrinsic motivation (three items), perception of work value (four items), societal motivation (four items), and financial motivation (four items).

Regression models: in this study, Multivariate Tobit regression models were performed to explore the association of work motivation among health care workers and demographic characteristics, workplace characteristics, and the impact of COVID-19 on work. Moreover, Multiple ordered logistic regression models were carried out to examine the association of change in job motivation, job satisfaction, commitment to the current job, and the above explanatory factors among health workers. Stepwise forward selection strategies were used with a log-likelihood ratio test at a *P*-value of 0.2 to find the minimal model. The *P*-value (*P* < 0.05) was considered statistically significant.

## RESULTS

**Table 1** demonstrated the demographic characteristics of respondents. Over half of the participants were female (56.5%), around two-thirds (62.7%) lived in cities and 60.1% resided in the Northern region. 72.3% were married. The majority of health officials responded (42.3%) had an average household income of 5-10 million VND per month.

**Table 1:** Demographic characteristics of respondents

Characteristics	n	%
**Total**	939	100.0	
Age group			
*21-30*	355	37.8	
*31-40*	460	49.0	
*≥40*	124	13.2	
Gender			
*Male*	408	43.5	
*Female*	530	56.5	
Education level			
*Intermediate / college*	168	17.9	
*Graduated*	420	44.7	
*Post-graduated*	351	37.4	
Area			
*City*	589	62.7	
*Town*	154	16.4	
*Rural, mountainous*	196	20.9	
Region			
*Northern*	563	60.1	
*Central*	233	24.9	
*Southern*	141	15.0	
Housing status			
*Private house with parents*	563	60.0	
*Private house without parents*	192	20.4	
*Rented house or others*	184	19.6	
Marital status			
*Single / divorced / widowed*	241	25.7	
*Married*	698	74.3	
Main income/mo			
*Under 5 million VND*	243	25.9	
*5-10 million VND*	533	56.8	
*10 million VND or above*	162	17.3	
Monthly household income per capita			
*Under 5 million VND*	372	39.7	
*5-10 million VND*	397	42.3	
*10 million VND or above*	169	18.0	

VND – Vietnamese dong

**Table 2** demonstrated the work characteristics and impacts of COVID-19. The majority of participants (70.9%) indicated that they were working with a contract of public employees. Just over half (54.1%) were medical doctors, and nurses/midwives accounted for the second-largest proportion of participants (21.5%). The majority of participants faced an increase in work-related stress and fatigue.

**Table 2:** Work characteristics and impacts of COVID-19 on respondents

Characteristics	n	%
Type of workplace		
*General hospital*	421	44.8
*Specialized / private hospital*	168	17.9
*CDC / medical centre / public health station*	213	22.7
*University hospital*	77	8.2
*Other*	60	6.4
Level of workplace		
*Central line*	201	21.4
*Province / city line*	347	37.0
*District line*	272	29.0
*Others*	119	12.7
Specialty		
*Medical doctor*	508	54.1
*Nurse / midwife*	202	21.5
*Technician/administrative staff*	113	12.0
*Others*	116	12.4
Contract status		
*Civil servant*	666	70.9
*Indefinite-term labour contract*	151	16.1
*Others*	122	13.0
Work experience		
*Under 5 years*	272	29.0
*5 years to under 10 years*	354	37.7
*Above 10 years*	313	33.3
Working seniority		
*Under 5 years*	358	38.1
*5 years to under 10 years*	325	34.6
*Above 10 years*	256	27.3
Number of duties per week		
*None*	251	26.7
*1 days*	226	24.1
*2 days*	254	27.1
*3 days and above*	208	22.2
Working time		
*Under 8 hours*	130	13.8
*8 hours*	417	44.4
*9-10 hours*	213	22.7
*11-12 hours*	105	11.2
*Above 12 hours*	74	7.9
Part-time job		
*None*	752	80.1
*One*	154	16.4
*Two and more*	33	3.5
The period of participating in the fight against the COVID-19 pandemic		
*None*	224	23.9
*1-3 months*	324	34.5
*3-12 months*	151	16.1
*Above 12 months*	240	25.6
	**Mean**	**SD**
Recent changes in work (range: 1-5)		
*Daily work intensity*	2.9	1.0
*Level of work-related stress and fatigue*	3.0	1.0
*Health risks caused by work*	2.9	1.1
*The community's stigma with the work I'm doing*	2.2	1.1
*Your ability to endure and cope with external work pressures*	2.5	1.1
*Process and professionalism of routine work*	2.7	1.0
*Complexity in coordination between colleagues, and between departments*	2.6	1.1
*New knowledge and skills for work*	2.8	1.0
*Ability to complete assigned tasks*	2.5	1.1
*Ability to ensure safe means of work*	2.6	1.1

CDC – centers for disease control and prevention, SD – standard deviation

From the data in **Table 3**, it is apparent that most participants tend to keep their jobs and only a few people have the intention to change jobs (2.5% certainly not, 3.6% very little possibility of commitment to the current job). There were 39.4% of participants decreased job satisfaction (16.9% dramatically decrease, 22.5% slightly decrease) and 49.6% of those decreased job motivation (23.9% dramatically decrease, 25.7% slightly decrease).

**Table 3:** Characteristics of commitment to the current job, change in job satisfaction, and motivation of participants

Characteristics	n	%
Commitment to the current job		
*Certainly not*	23	2.5
*Very little possibility*	34	3.6
*Unclear*	202	21.5
*Very much possibility*	331	35.3
*Definitely yes*	349	37.2
Change in job satisfaction		
*Dramatically decrease*	159	16.9
*Slightly decrease*	211	22.5
*Unchanged*	383	40.8
*Increase*	83	8.8
*Significantly increase*	103	11.0
Change in job motivation		
*Dramatically decrease*	224	23.9
*Slightly decrease*	241	25.7
*Unchanged*	357	38.0
*Increase*	54	5.8
*Significantly increase*	63	6.7

**Table 4** provides the construct reliability and validity of the burnout factor from medical workers. Four dimensions were classified namely “intrinsic motivation”, “perception of work value”, “societal motivation” and “financial motivation”. Cronbach’s alphas range from 0.85 to 0.91 and are stable across domains.

**Table 4:** Factor loadings of work motivation scale for health workers

Items (range: 0-10)	Mean (SD)	Intrinsic motivation	Perception of work value	Societal motivation	Financial motivation
1. Because I enjoy doing what I do at work every day.	6.9 (2.5)	0.73			
2. Because I enjoy my work tasks.	7.1 (2.3)	0.81			
3. Because the work that I do is very interesting.	6.6 (2.5)	0.76			
4. Because being a health worker is a fundamental part of who I am	7.6 (2.4)		0.64		
5. Because my work is extremely important for my patients	7.9 (2.3)		0.64		
6. Because I want to make a difference in people’s life	7.0 (2.6)		0.59		
7. In order to feel good about myself	7.6 (2.3)		0.58		
8. Because my reputation depends on my work.	6.2 (3.0)			0.68	
9. Because of the appreciation I receive from my patients and the community	7.0 (2.5)			0.68	
10. So I do not let my team down	7.6 (2.3)			0.51	
11. Because my supervisor recognizes and appreciates me	6.7 (2.6)			0.65	
12. Because of the benefits that come with my job	6.2 (2.8)				0.51
13. In order to be able to provide for my family	7.1 (2.7)				0.82
14. Because of the financial security my job provides me with	6.7 (2.7)				0.82
15. In order to earn money	6.6 (2.9)				0.73
Floor (%)		2.3	0.8	1.4	1.4
Ceiling (%)		12.5	13.0	9.1	11.9
Reliability					
Cronbach’s alpha		0.91	0.86	0.85	0.87
Domains scores					
Mean		27.5	30.0	27.6	26.6
SD		9.0	8.0	8.7	9.4

SD – standard deviation

**Table 5** presents the Coefficient (Coef.), and 95% confidence interval (CI) from Tobit and Ordered logistic regression analysis. Statistically significant factors associated with motivation in three domains: demographic, workplace characteristics, and impacts of COVID-19 were: age, education level, marital status, region of work, income per month, contract status, and changes in work. Participants in the central region are likely to be more motivated than those in the north in all four factors (Coef. = 2.11; 2.90). Health professionals in the age group over 40 and from 31-40 tend to be more motivated to work than the age group 21-30 in all four components (Coef. = 1.14; 5.75). Those who had a low level of adaptability to external work pressure tended to be less motivated (Coef. = 3.70; -2.3).

**Table 5:** Factors associated with motivation among health professionals

Variables	Intrinsic motivation	Perception of work value	Societal motivation	Financial motivation
	**Coef. (95% CI)**	**Coef. (95% CI)**	**Coef. (95% CI)**	**Coef. (95% CI)**
**Demographic information**				
Age group (21-30 – ref)				
*31-40*	1.88† (0.24-3.52)	2.13‡ (0.80-3.45)	1.91‡ (0.49-3.34)	1.14 (-0.42,2.69)
*≥40*	5.35‡ (2.58-8.13)	5.47‡ (3.53-7.41)	5.75‡ (3.67-7.82)	3.54‡ (1.06-6.01)
Gender (male – ref)				
*Female*	1.12 (-0.41,2.66)			0.97 (-0.41,2.36)
Education level (intermediate / college – ref)				
*Graduated*	-3.60‡ (-5.81,-1.39)	-4.61‡ (-6.41,-2.82)	-3.63‡ (-5.35,-1.92)	-4.37‡ (-6.31,-2.44)
*Post-graduated*	-3.12‡ (-5.49,-0.76)	-3.96‡ (-6.05,-1.88)	-2.37† (-4.19,-0.56)	-3.49‡ (-5.59,-1.39)
Marital status (single / divorced / widowed – ref)				
*Married*		-1.45† (-2.87,-0.03)	-1.43* (-2.95,0.09)	
Housing status (private house with parents – ref)				
*Private house without parents*				-1.29 (-3.00,0.42)
*Rented house or others*				0.84 (-0.94,2.61)
Area (city – ref)				
*Town*			1.64* (-0.04,3.32)	
*Rural, mountainous*			0.71 (-0.86,2.28)	
Region (Northern – ref)				
*Central*	2.11† (0.18-4.03)	2.90‡ (1.52-4.28)	2.22‡ (0.76-3.69)	2.51‡ (0.86-4.17)
*Southern*	2.06* (-0.13,4.25)	1.47* (-0.18,3.13)	1.46 (-0.32,3.25)	1.73* (-0.28,3.74)
The main income per month (under 5 million VND – ref)				
*5-10 million VND*	1.86† (0.05,3.67)			2.65‡ (1.00-4.30)
*10 million VND or above*	4.13‡ (1.42,6.83)			4.20‡ (1.81-6.59)
*Workplace information*				
Level of the workplace (central line – ref)				
*Province / city line*	-2.24† (-4.46,-0.02)			
*District line*	-2.82† (-5.19,-0.44)			
*Others*	-1.10 (-3.94,1.73)			
Specialty (medical doctor – ref)				
*Nurse / midwife*		0.66 (-1.16,2.49)		
*Technician / administrative staff*		-3.04‡ (-4.92,-1.15)		
*Others*		-0.60 (-2.43,1.23)		
Contract status (civil servant – ref)				
*Indefinite-term labour contract*				-0.68 (-2.55,1.18)
*Others*				4.01‡ (1.85-6.18)
Working time (under 8 hours – ref)				
*8 hours*		0.57 (-1.15,2.30)		
*9-10 hours*		0.54 (-1.38,2.47)		
*11-12 hours*		1.67 (-0.61,3.94)		
*Above 12 hours*		3.49‡ (0.96-6.03)		
Impact of COVID-19				
The period of participating in the fight against the COVID-19 pandemic (none – ref)				
*1-3 months*				-0.17 (-2.04,1.71)
*3-12 months*				-1.57 (-3.80,0.67)
*Above 12 months*				-2.12† (-4.13,-0.12)
Recent changes in work (unit: per score)				
*The community's stigma with the work I'm doing*	-1.50 (-3.40,0.39)	-0.93 (-2.32,0.45)		
*Your ability to endure and cope with external work pressures*	-3.70‡ (-6.32,-1.07)	-2.91‡ (-4.67,-1.15)	-2.38† (-4.19,-0.57)	-2.98‡ (-4.79,-1.16)
*Process and professionalism of routine work*	7.18‡ (3.86-10.49)	3.77‡ (1.65-5.89)	3.57‡ (1.36-5.79)	
*Complexity in coordination between colleagues, and between departments*	-2.26 (-5.20,0.69)			
*Ability to complete assigned tasks*	-2.71† (-4.96,-0.46)			

CI – confidence interval, VND – Vietnamese dong

*P < 0.01, †*P* < 0.05, ‡*P* < 0.1.

**Table 6** presents factors associated with changes in job satisfaction, motivation, and commitment. Variables most correlated were: age, marital status, specialty, and community stigma. Married people tended to be more committed to their current job (odds ratio (OR) = 1.66; 95% CI = 1.23-2.25). Highly internally motivated people were more likely to commit to work (“intrinsic motivation”: OR = 1.09; 95% CI = 1.07-1.12) and those with more than 10 years of experience recorded higher job satisfaction and motivation compared to those with less than five years of experience.

**Table 6:** Factors associated with changes in job satisfaction, motivation, and commitment

Variables	Change in job satisfaction (from 1 “dramatically decrease” to 5 “significantly increase”)	Change in job motivation (from 1 “dramatically decrease” to 5 “significantly increase”)	Commitment to the current job (from 1 “certainly not” to 5 “definitely yes”)
	**OR (95% CI)**	**OR (95% CI)**	**OR (95% CI)**
**Demographic information**			
Age group (21-30 – ref)			
*31-40*	0.64† (0.45-0.91)	0.74* (0.52-1.05)	
*≥40*	0.74 (0.42-1.30)	0.87 (0.49-1.55)	
Gender (male – ref)			
*Female*	1.31† (1.01-1.70)		
Education level (intermediate / college – ref)			
*Graduated*	1.62† (1.08-2.43)	1.17 (0.82-1.68)	0.57† (0.37-0.89)
*Post-graduated*	1.23 (0.77-1.98)	0.73* (0.50-1.06)	0.59† (0.36-0.97)
Marital status (single / divorced / widowed – ref)			
*Married*			1.66‡ (1.23-2.25)
Region (Northern – ref)			
*Central*			1.02 (0.74-1.42)
*Southern*			0.54‡ (0.37-0.78)
The main income per month (under 5 million VND – ref)			
*5-10 million VND*		1.38† (1.01-1.90)	
*10 million VND or above*		1.22 (0.76-1.95)	
Monthly household income per capita (Under 5 million VND – ref)			
*5-10 million VND*		0.74† (0.56-0.98)	1.39† (1.04-1.85)
*10 million VND or above*		0.70* (0.47-1.02)	1.08 (0.75-1.57)
*Workplace information*			
Level of the workplace (central line – ref)			
*Province / city line*	0.75 (0.53-1.07)	0.67† (0.48-0.95)	1.27 (0.88-1.84)
*District line*	0.81 (0.55-1.18)	0.66† (0.46-0.95)	0.90 (0.61-1.35)
*Others*	1.19 (0.75-1.90)	1.04 (0.67-1.60)	1.27 (0.77-2.09)
Specialty (medical doctor – ref)			
*Nurse / midwife*	1.86‡ (1.21-2.86)		1.75† (1.14-2.69)
*Technician/administrative staff*	1.41 (0.91-2.18)		1.20 (0.77-1.86)
*Others*	1.76‡ (1.15-2.68)		2.10‡ (1.35-3.28)
Working experience (under 5 years – ref)			
*5 years to under 10 years*	1.43* (1.00-2.05)	1.40* (0.98-2.01)	
*Above 10 years*	2.44‡ (1.49-3.99)	2.64‡ (1.65-4.21)	
Contract status (civil servant – ref)			
*Indefinite-term labour contract*	0.66† (0.47-0.93)		0.79 (0.56-1.12)
*Others*	0.78 (0.51-1.18)		0.52‡ (0.35-0.78)
Part-time job (none – ref)			
*1*			0.70† (0.50-0.98)
*2 and more*			0.74 (0.36-1.50)
Impact of COVID-19			
Working time (under 8 hours – ref)			
*8 hours*	0.94 (0.64-1.38)		
*9-10 hours*	0.66* (0.43-1.01)		
*11-12 hours*	0.98 (0.58-1.64)		
*Above 12 hours*	1.08 (0.60-1.95)		
Recent changes in work, range: 1-5 (unit: per score)			
*The community's stigma with the work I'm doing*	0.72† (0.54-0.95)	0.69‡ (0.53-0.90)	0.61‡ (0.44-0.84)
*Your ability to endure and cope with external work pressures*			1.34 (0.89-2.01)
*New knowledge and skills for work*	0.71 (0.45-1.12)		
*Ability to ensure safe means of work*			0.50‡ (0.33-0.77)
Motivation (unit: per score)			
*Intrinsic motivation*	1.07‡ (1.05-1.09)	1.09‡ (1.07-1.12)	1.09‡ (1.07-1.12)
*Perception of work value*		1.04‡ (1.01-1.06)	1.03† (1.01-1.05)
*Societal motivation*		0.98† (0.96-1.00)	
*Financial motivation*	1.02† (1.00-1.03)		

CI – confidence interval, VND – Vietnamese dong

*P < 0.01, †*P* < 0.05, ‡*P* < 0.1.

## DISCUSSION

Our results suggest a serious deterioration of work motivation among health workers in Vietnam during the exhaustive 2021 outbreak of COVID-19 and point out the individual, environment, and interpersonal factors associated with such change. Notably, besides conventional factors such as workloads or administrative level of the workplace, variables of health-specific and COVID-19-specific context such as adaptability to work pressure or work safety also have major impacts on motivation.

Our results show statistically insignificant differences between levels of intrinsic and extrinsic motivation among health care workers. This dynamic is especially notable as, before COVID-19, financial incentives were often considered the biggest driver of performance [24-26]. Even when it is not explicitly stated, various health reform strategies have relied on money as the key motivator [27]. A reduction in the gap between financial motives and other forms of motivations suggests that either intrinsic motives have increased in importance, or financial motivators have faded in effect. Our results suggest the latter is true. In this study, while it is still evident that a higher income level correlates with a higher level of motivation, an opposite trend emerged with COVID-19-specific variables. The longer health workers participate in COVID-19 tasks, the less they are motivated by financial incentives: COVID-19 health providers for above a year recorded critically low levels of financial motivation compared to those who did not participate in fighting COVID-19. This indicates that the effect of financial motives diminishes over time and after around one year, it will have no considerable attraction on health care workers. At the moment, Vietnamese policymakers still resort to financial compensation for health care officials as a form of encouragement [28,29], and this pattern of encouragement must be adjusted. Various past studies have pointed out that financial incentives, if overused, are not only unproductive but will also become counter-productive eventually [30,31]. Therefore, policymakers should start looking at other sources of motivation to direct available resources instead of only focusing on salary raises.

The main implication of our study is that health care managers and policymakers resolve issues about the intrinsic motivations of health care workers immediately. The two most outstanding factors in our study are workers’ ability to work under pressure and the professionalism of routine work. A low level of adaptation to stress during COVID-19 is evident in our research and many others about health care workers worldwide. Healthcare delivery, even in its normal context, is heavily labour-intensive, meaning it is critically dependent on the participation of workers. Under the influence of the pandemic, the role of health care workers was even more amplified as they had to singlehandedly cater to the need of a whole infected nation under lockdown. Most importantly, the surge in health care workload was immediate and intense, leaving very little to no time for workers to adjust. Disruption of the existing coordinative work pattern and problems around professionalism of routine work also arose from these sudden changes. Therefore, as the surge in new cases has temporarily ceased, the immediate suggestion is that authorities introduce small breaks for overworked health care workers and develop more logical shift patterns for the next post-peak period. Mental support and stress relieving lessons should also be made available regularly to tackle remaining psychological issues and improve the adaptability to stress of health care workers for other health crises. In further future, health care managers should develop a response protocol or an emergency work distribution system that allows changes in workload to be introduced more gradually.

The ultimate goal of work motivation improvement is to encourage the commitment of health care workers toward a stable workforce. Our findings suggest that the factor most correlated with work commitment was the community’s stigma. Throughout the pandemic, social media has been the most imminent tool in directing attention and shaping public perceptions [32,33]. Although the power of media allowed for various mental interventions and positive information, it also fuelled harmful stereotypes and furthered the discrimination toward health care workers [34,35]. Therefore, sources of information must be managed to reduce the spread of dangerous, incorrect news about health care workers. Moreover, a better retention rate usually goes alongside a higher level of self-perception of work value, more specifically the perceived meaningfulness of one’s contribution to society. In the same attempt to control social media output, authorities should also utilize their power to promote the importance of health care workers and express appreciation to society for the health workforce.

The main strength of this study lies in its sample size. We were able to record and analyse a total of 939 responses from all demographic backgrounds and different levels of the health system. However, certain limitations exist. First, the cross-sectional study design does not allow for the inference of causal relationships. Second, as the study was carried out during the November peak of COVID-19, health care workers were generally overloaded and many could not participate in the online survey. Our study, therefore, could not be representative of the whole frontline workforce. Lastly, data collected through self-reporting may be subject to personal bias. Nonetheless, this study is among the largest efforts to measure work motivation during COVID-19 and is able to identify existing problems within the health system as well as signify important implications for policy adjustments.

## CONCLUSION

The fourth and largest outbreak of COVID-19 in Vietnam has moved to its post-peak period, but our fight against COVID-19 is far from ending. It is now time for us to resolve the remaining problems within the health system such as the deterioration of work motivation, and rethink our approaches for future outbreaks. Our study highlighted the need to promote intrinsic motivations among health care workers as well as provide suggestions for the healthy and long-term development of the workforce. Ultimately, this paper is a reminder for Vietnam to recognize and honour the sacrifices of the health care workforce after three years of incredibly selfless service.
